# Unprecedented plant species loss after a decade in fragmented subtropical Chaco Serrano forests

**DOI:** 10.1371/journal.pone.0206738

**Published:** 2018-11-28

**Authors:** Ramiro Aguilar, Ana Calviño, Lorena Ashworth, Natalia Aguirre-Acosta, Lucas Manuel Carbone, Guillermo Albrieu-Llinás, Miguel Nolasco, Adrián Ghilardi, Luciano Cagnolo

**Affiliations:** 1 Instituto Multidisciplinario de Biología Vegetal, Universidad Nacional de Córdoba, Consejo Nacional de Investigaciones Científicas y Técnicas, Córdoba, Argentina; 2 Laboratorio Nacional de Análisis y Síntesis Ecológica, Escuela Nacional de Estudios Superiores, Unidad Morelia, Universidad Nacional Autónoma de México, Antigua Carretera a Pátzcuaro, Morelia, México; 3 Facultad de Ciencias Agropecuarias, Universidad Nacional de Córdoba, Córdoba, Argentina; 4 Laboratorio de Arbovirus, Instituto de Virología "Dr. Vanella", Facultad de Ciencias Médicas, Universidad Nacional de Córdoba, CONICET, Córdoba, Argentina; Lund University, SWEDEN

## Abstract

Current biodiversity loss is mostly caused by anthropogenic habitat loss and fragmentation, climate change, and resource exploitation. Measuring the balance of species loss and gain in remaining fragmented landscapes throughout time entails a central research challenge. We resurveyed in 2013 plant species richness in the same plots of a previous sampling conducted in 2003 across 18 forest fragments of different sizes of the Chaco Serrano forest in Argentina. While the area of these forest remnants was kept constant, their surrounding forest cover changed over this time period. We compared plant species richness of both sampling years and calculated the proportion of species loss and gain at forest edges and interiors. As in 2003, we found a positive relationship between fragment area and plant richness in 2013 and both years showed a similar slope. However, we detected a net decrease of 24% of species’ richness across all forest fragments, implying an unprecedentedly high rate and magnitude of species loss driven mainly by non-woody, short-lived species. There was a higher proportion of lost and gained species at forest edges than in forest interiors. Importantly, fragment area interacted with percent change in surrounding forest cover to explain the proportion of species lost. Small forest fragments showed a relatively constant proportion of species loss regardless of any changes in surrounding forest cover, whereas in larger fragments the proportion of species lost increased when surrounding forest cover decreased. We show that despite preserving fragment area, habitat quality and availability in the surroundings is of fundamental importance in shaping extinction and immigration dynamics of plant species at any given forest remnant. Because the Chaco Serrano forest has already lost 94% of its original cover, we argue that plant extinctions will continue through the coming decades unless active management actions are taken to increase native forest areas.

## Introduction

The spatial distribution and assembly of biodiversity on earth have always been under stochastic and deterministic changes triggered by natural processes of varied sources (e.g., glaciations, geological transformations, volcanic activity, continental drift). Such community dynamics are governed by extinction and immigration of species throughout timescales of decades to millennia and across local to regional spatial scales [1]. Today, however, biodiversity loss is mostly caused by anthropogenic habitat loss and fragmentation, climate change, transcontinental species introductions, and resource exploitation [1, 2]. In particular, land use changes imposed by the expansion of urban and agricultural frontiers are predicted to drive the most significant effects on biodiversity throughout this century [2–4].

The concept of extinction debt (sensu [5]) implies that extinction of species can take place with a considerable delay after the event of habitat loss or any other deterministic change has occurred. Several factors may influence the occurrence and extent of extinction debts as well as the time elapsed until reaching a new equilibrium within a community (i.e., relaxation time [6]). The magnitude and spatial scale of habitat loss will determine the degree of connectivity of remaining habitat at the landscape level, affecting the persistence and viability of certain species [6, 7]. Extinction debts are more likely to be paid off more slowly in moderately fragmented landscapes with high connectivity or large patches of remaining habitat nearby and more quickly in highly fragmented landscapes, with smaller remaining forest fragments and low habitat cover in their surroundings [6, 8]. On the other hand, within remaining forest fragments, some species will be more susceptible to undergo delayed extinction. For example, long-lived species are particularly likely to face delayed extinctions [6, 9]. In the case of plants, woody species such as trees and shrubs are long-lived and thus persist longer in chronically small and isolated forest fragments and also have several reproductive opportunities throughout time to yield and recruit successful progeny (e.g. [10, 11]). In contrast, non-woody species such as grasses, herbs, and herbaceous vines have higher turnover rates as their lifespan is, in general, much shorter than woody species [12]. As a result, short-lived non-woody plant species are likely to pay off faster the extinction debt than long-lived woody species [6, 10, 11].

Studies assessing the phenomenon of an extinction debt in plant communities show contrasting results (reviewed by [6, 7]), and the vast majority were conducted in European semi-natural grasslands and other temperate northern-hemisphere ecosystems, which have been under large-scale human pressure for centuries (reviewed by [13]). No studies assessing delayed extinctions have yet been conducted in subtropical forests of South America. The Gran Chaco, the largest seasonally dry forest in the southern hemisphere, has been understudied and thus underrepresented as a key ecosystem in South America [14]. This forest has been subjected to strong human pressure holding the highest deforestation rates worldwide over the past recent decades [14–18]. Most of the studies evaluating delayed extinctions have used two main approaches: (i) studies based on old maps that document relationships between past landscape patterns and present-day species diversity (e.g. [19–21]); and (ii) studies based on present day stable and unstable landscapes (e.g., [9, 22]). Only a few studies were able to sample plant species richness within the same fragmented landscape at two different times to assess the occurrence of delayed extinctions [23–26].

One potential drawback of previous attempts to measuring delayed extinctions is the failure of simultaneously accounting for potential species immigration, which may also take place in fragmented habitats [1, 25–27]. For example, species loss may be offset by increased immigration of pioneer species that take advantage of vacant niches that are available in remaining forest fragments, especially at their edges [28–30]. Thus, incorporating the simultaneous assessment of species extinction and species immigration may enable us to approach the paradigm of continuous changing dynamics of biodiversity in current landscapes [1, 27]. Measuring the transient dynamics of species loss and gain in current remaining fragmented landscapes entails a central research challenge, with key implications for biological conservation and the preservation of ecosystem services provided by biodiversity [1, 4, 27, 31].

Here, we aim to evaluate plant species richness in a fragmented landscape to measure species loss and gain after a decade. To accomplish this, we resurveyed in 2013 a previous sampling of plant species conducted in 2003 [32] across 18 forest fragments of different sizes of the Chaco Serrano forest in Argentina. All these forest fragments maintained their areas constant throughout this time period. Back in 2003, Cagnolo et al. [32], found a positive relationship between the area of forest fragments and plant species richness. Because small forest fragments may show higher rates of species extinctions through demographic and stochastic processes, the slope of the species-area relationship should be steeper in 2013 than that found in 2003. In other words, there will be more lost plant species in smaller than larger forest fragments and such extinctions will mostly involve non-woody short-lived species. However, while the area of these forest fragments was kept constant, the habitat surrounding them may have undergone changes throughout this time, affecting their colonization and extinction rates (e.g. [33, 34]). An increased amount of habitat around a forest fragment may delay the payment of an extinction debt whereas decreased habitat amount surrounding a forest fragment after ten years may accelerate the payment. Thus, here we raise the following questions: (i) What is the balance of species loss and gain at the landscape level after a decade? (ii) Are species loss and gain associated to particular forest fragment characteristics (fragment area, edge-interior status, and percent change of surrounding forest cover after ten years), (iii) and/or species life form (woody versus non-woody)?

## Materials and methods

### Study area

The Chaco Serrano forest of Central Argentina has suffered a major loss over the past 40 years, leading to a reduction of 94% of its original area [15]. Today, the 6% of native forest left is presented as fragments of different sizes containing an area-specific subset of the original flora and fauna [15, 32, 35–37]. In Córdoba province, the Chaco Serrano ranges in elevation from 400 to 1300 m above sea level with an annual rainfall of 750 mm that is concentrated mostly in the warm season (October–April), and mean minimum and maximum temperatures of 10°C and 26°C respectively [36]. The predominant vegetation is a xerophytic subtropical forest, characterized by closed and open forests depending on grazing pressures and fire recurrence [36], and it is currently restricted to isolated forest fragments within an intensely managed matrix [15, 16]. The tree layer is 8–15 m high and dominated by *Lithraea molleoides* (Vell.) Engl., *Celtis ehrenbergiana* (Klotzch) Liebm., *Acacia caven* (Molina) Molina, *Condalia* spp., and *Zanthoxylum coco* Gillies ex Hook. f. and Arn. [36]. The growth forms most represented in Chaco Serrano, however, are annual and perennial herbs and graminoids, comprising 63% of the species [36].

**Fig 1:**
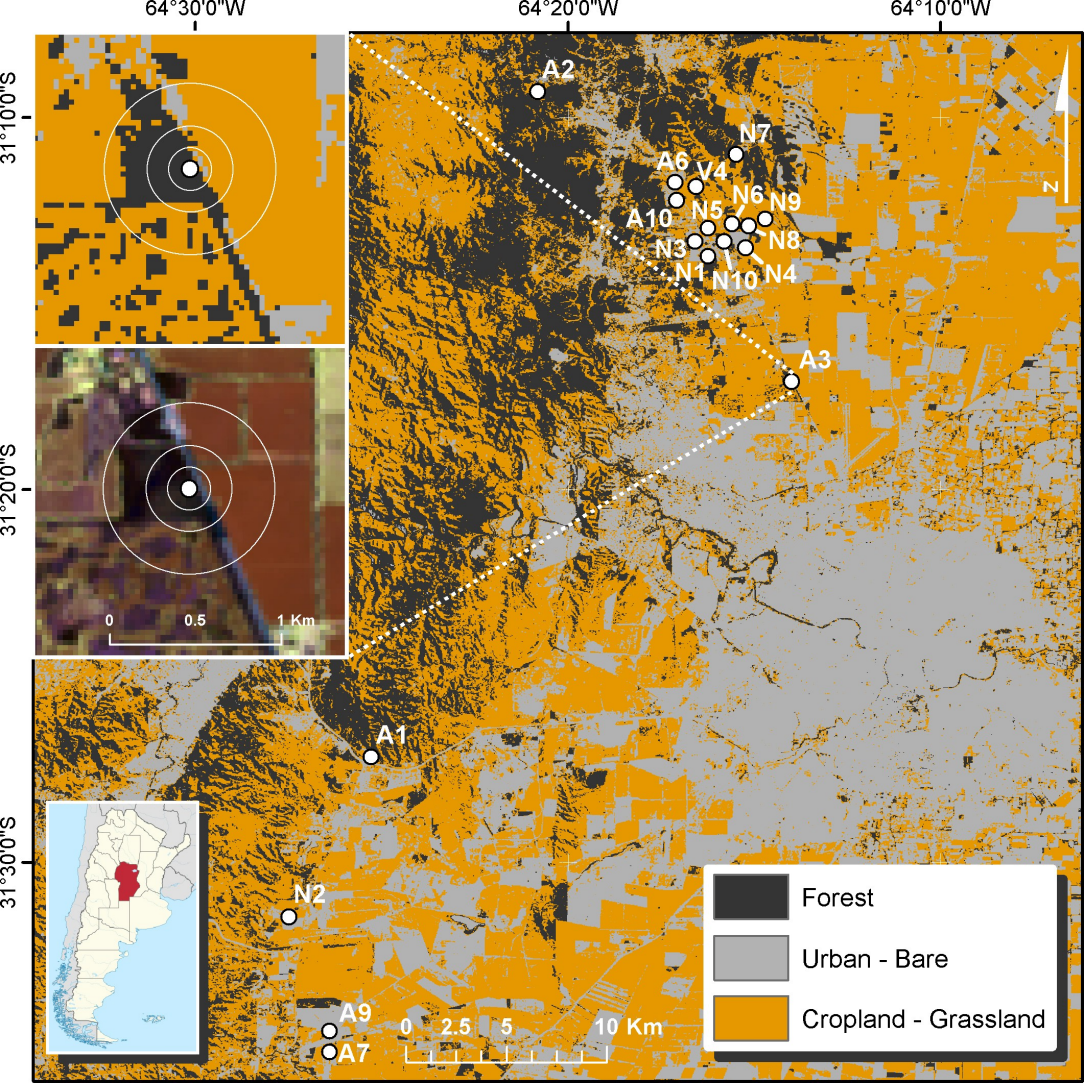
Map of the study sites. The map is showing 18 Chaco Serrano forest fragments resurveyed in 2013. We show the expanded view of a single forest patch with the three buffer distances of 250, 500 and 1000m used to calculate the percent change in forest cover after a decade with land-cover classifications using LANDSAT imagery.

In 2003, Cagnolo et al. [32] evaluated a large area between 31°05’–31°35’S and 64°10’–64°30’W, using LANDSAT digital satellite images (Fig 1). After assessing accessibility to different sites, with granted permission by private owners, and trying to maintain isolation among fragments and keep matrix characteristics relatively constant, they selected 19 forest fragments with areas ranging from 0.13 ha to approximately 1000 ha [32]. These forest fragments were created during the 1970s when vast areas of Chaco Serrano forest were transformed into agricultural and urban areas [15, 16, 37]. The studied forest fragments were located at a minimum distance that ranged between 75–200 m of any other forest fragment (Fig 1). All forest fragments are embedded in agricultural matrices of wheat in winter and soybean or maize in summer, and all of them, including the largest tracts of forests, experience cattle grazing. We were able to resample plant species richness in 18 forest fragments (Fig 1), as one of them disappeared by 2013 as a result of the expansion of urbanization.

### Floristic data

Back in 2003, Cagnolo et al. [32] established two 20 x 25 m sampling plots in each forest fragment. One of these sampling plots was set at 5 m inside the forest and the other one at the interior (50 m from the edge in the case of large fragments and at the geometric center in fragments <4 ha) of each forest fragment. With the coordinates from a Global Positioning System device used in 2003 and the help of Dr. Cagnolo in the field, we were able to resurvey the same sampling plots used by Cagnolo et al. [32]. Each of these landmarks represents the average coordinates taken from multiple GPS readings. Thus, they have an accuracy of < 1m. We also followed the same sampling protocol used by Cagnolo et al. [32] within the same sampling months (April and May). As in 2003, three operators conducted the surveys at each sampling plot: while two of them walked along the sampling plot surveying all vascular plant species present within 10 m width each, a third operator took notes on their observations. The size of sampling plots represents the minimal area needed to include almost all the species present, and they were determined by using species accumulation curves [38, 39].

All the species were collected and deposited in the Botanical Museum of the National University of Córdoba (CORD). We also thoroughly revised the taxonomic identification of the plant species list obtained in 2003 by Cagnolo et al. [32], as these original specimens were deposited in the Botanical Museum (CORD). Both species lists were unified with updated taxonomical classification following the Flora Argentina Catalogue [40]. After eliminating taxonomic synonymies and correcting misidentified taxa, we rectified the original 2003 plant list from 253 species reported in Cagnolo et al. [32] to 229 unique plant species (S1 Table).

To determine the relative life form of plant species in a broad sense, we categorized species as woody or non-woody, based on the evidence that woody plant species have on average a projected life span more than four times as long as non-woody plants [12]. Thus, woody species such as trees, shrubs and some vine species were considered to be long-lived species, whereas non-woody herbs, grasses, ferns and some vines were considered short-lived species [12] (S1 Table).

### Calculation of land cover metrics

Two images from the multispectral satellites Landsat 5 and Landsat 8 [41] (path/row: 229/82), acquired on 17 December 2003 and 13 January 2014 respectively, were used to generate a land cover classification. The level 1 raster images were downloaded as top-of-atmosphere reflectance values from the Google Earth Engine platform (https://earthengine.google.com). Briefly, a supervised classification based on the support vector machine (SVM) algorithm [42] was performed to classify the area under study obtaining 5 land cover classes: high vegetation (bushes and trees), urban buildings, bare soil, low vegetation (grass/crops), and water. The SVM algorithm was run using the radial basis function (RBF) kernel and optimized C and γ parameters. The classification was executed using the open source Orfeo Toolbox in the QGIS 2.14 free software [43]. In order to assess the classification accuracy, error matrixes were calculated and the overall accuracy and Kappa indexes were then derived from the error matrixes [44]. Reference data were obtained from a combination of ground control points, and the visual analysis of the Landsat images. The land cover statistics plugin (LecoS) [45] was used in QGIS 2.14 to calculate the total and proportional areas covered by high vegetation. To this end, a vector corresponding to circular buffer areas of 250m, 500m, and 1000m diameter, was overlaid to each classified map, and used to extract the mentioned metrics in both 2003 and 2013. We then calculated the percent change in forest cover (i.e., high vegetation) around each studied forest fragment after ten years as follows: ((forest cover 2013—forest cover 2003) / forest cover 2003)*100. Thus, negative values imply a loss of forest cover in the buffer areas whereas positive values imply an increase of forest cover after ten years. Another vector was constructed covering the entire studied area, and it was used to extract the same metrics at a regional scale. We also calculated the percent change in forest cover after ten years at this broader regional spatial scale.

### Data analysis

A global matrix with the species presence-absence data per sampling plot and year was first used to compare the overall species richness and the species richness-area relationships between 2003 and 2013. A similar matrix, but categorizing species by life form was used to compare the species richness and richness-area relationship between 2003 and 2013 for woody and non-woody species. We further merged the data sets into a third matrix by combining both sampling years for each species entry. With this combined matrix of species presence-absence data, we calculated the proportion of lost and gained species categorized by their life form (woody and non-woody) at each of the 18 forest fragments and at each position within forest fragments (edge, interior) from 2003 to 2013. In order to standardize the proportions of lost and gained species by species richness within each life form category we calculated them as follows: (a) proportion of lost woody (or non-woody) species as the number of woody (or non-woody) species present in the fragment in 2003 but absent in 2013 divided by the total number of woody (or non-woody) species registered in the forest fragment in 2003 and 2013; (b) proportion of gained woody (or non-woody) species as the number of woody (or non-woody) species absent in 2003 but present in 2013 divided by the total number of woody (or non-woody) species registered in the forest fragment in 2003 and 2013.

To test for changes in plant species richness from 2003 to 2013 we used generalized linear mixed effects models (*glmer* function from the *lme4* package [46]) with Poisson distribution and with forest fragment area (log10 transformed), position (edge, interior), year (2003, 2013), life form (woody, non-woody) as fixed factors, and site as a random factor. To analyze the proportion of lost and gained species between 2003 and 2013 we performed similar models (*glmer* function and binomial distribution) to test the main effects of: fragment area, percent change in forest cover surrounding each forest fragment after ten years (at 250m, 500m, and 1000m), position and lifespan including site as a random factor. In all cases we only included first and second-degree interactions. Removal of non-significant fixed effects and interactions was performed with 'bfFixefLMER_t.fnc' function and 'llrt' method for glmer models from the 'LMERConvenienceFunctions' package [47]. This function back-fits an initial glmer model based on p-values (α = 0.05) obtained from iterative log-likelihood ratio testing [47]. In all cases, we report the z values from the reduced model. All analyses were performed in R environment [48]. Predicted probabilities of significant interaction terms were plotted with the ‘sjp.int’ function of the ‘sjPlot’ package [49]. This function plots the marginal effects of the interaction with the other covariates, and to do so the minimum and maximum values of the moderator variable were used to plot the interaction between the independent variable and the moderator.

## Results

In 2013 we recorded a total of 163 plant species throughout all forest fragments (combining all species of the two sampling plots per forest fragment; S1 Table). From the 229 plant species recorded in 2003, 91 species were lost in 2013, representing an overall 39% decrease in plant species richness after a decade. On the other hand, 25 out of 163 species were new in 2013 and not previously observed in 2003, implying a 15% species gain. As a result, there was a net loss of plant richness of 24% at the landscape level. The forest fragment that disappeared from the landscape in 2013 (1.5 ha) did not have unique plant species. At the patch level, every single resurveyed forest fragment, including the three largest tracts of forests, showed a systematic overall decrease in plant richness (Table 1). On average, 37 (± 10.7 s.d.) species were lost per fragment; whereas roughly 14 (± 5.7 s.d.) species on average per fragment were new in 2013, and 38 (± 9.8 s.d.) species remained in both sampling years (Table 1). At the regional level, there was a net decrease in forest cover from 2003 to 2013 of 14.38%.

**Table 1:** Balance of plant species richness after a decade. Species richness of sampled Chaco Serrano forest fragments in 2003 and 2013. The number of lost, gained, and remaining species per forest fragments after a decade were calculated from a species presence-absence database.

Fragment code	Area (Ha)	Richness 2003	Richness 2013	N° species lost	N° species gained	N° species remaining	Percent change (%) of surrounding forest cover after ten years		
							250m	500m	1000m
A1	~1000	126	73	73	20	53	-31.6	-32.2	-24.3
A2	~1000	88	86	33	31	55	-16.6	-13.0	-17.0
N7	~1000	93	61	46	14	47	-12.0	-8.2	-5.3
A9	116.16	83	52	48	17	35	-57.4	-63.9	-67.7
A3	29.53	98	71	42	15	56	-6.2	-5.5	+3.1
A7	13.77	63	42	35	14	28	+3.0	-11.4	0.00
N8	10.7	71	57	30	16	41	-2.9	-3.3	-11.2
N2	3.58	73	41	41	9	32	-26.3	-38.8	-17.9
N4	3.48	74	55	32	13	42	-3.3	0.00	-20.2
N9	3.24	72	46	37	11	35	-7.7	-7.4	+60.0
N1	2.89	72	38	39	5	33	-41.6	-57.1	-18.8
V4	2.22	70	53	30	13	40	-27.6	-23.5	-21.4
N3	1.25	62	41	33	12	29	-66.6	-63.6	-25.0
A6	1.14	61	42	27	8	34	-16.6	-69.2	-81.8
N5	0.78	57	42	28	13	29	+66.1	+21.0	-17.7
A10	0.7	55	41	30	16	25	-84.6	-96.4	-83.8
N10	0.57	72	50	33	11	39	0.00	-28.5	-8.9
N6	0.13	58	32	33	7	25	+33.3	+17.4	-18.8

**Fig 2:**
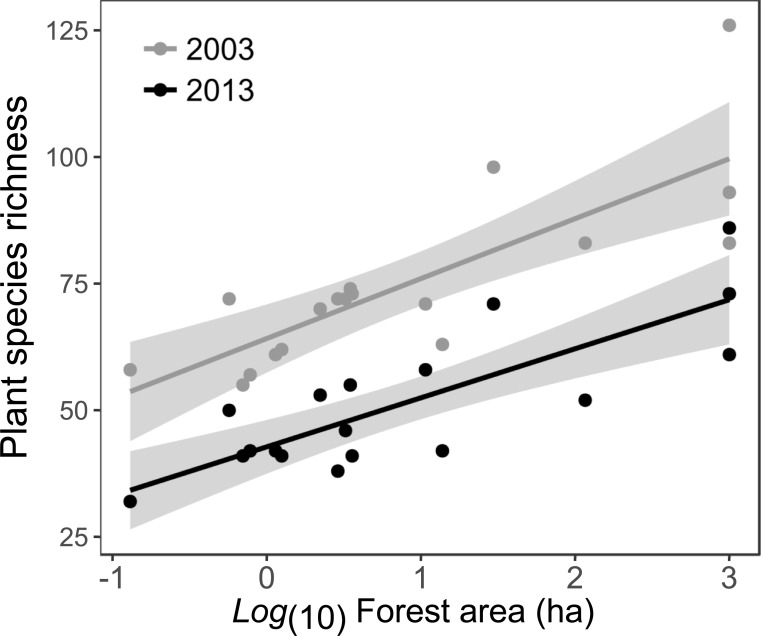
Plant species richness. Plant species richness-area relationships for the same Chaco Serrano forest fragments in 2003 (grey line and CI) and 2013 (black line and CI). CI = 95% Confidence interval.

**Fig 3:**
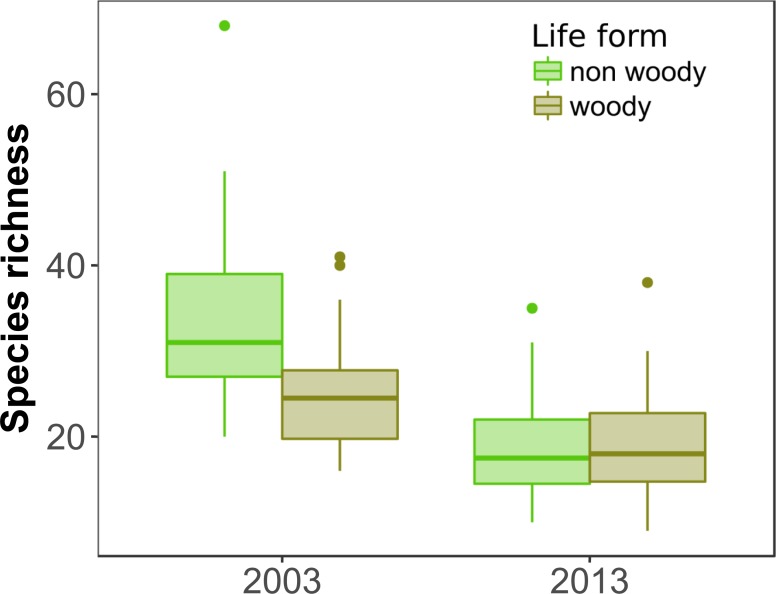
Richness of non-woody and woody plant species. Original surveys of plant species richness in 2003 and resurveys in 2013 for the same Chaco Serrano forest fragments. The bottom of each box is the 25th percentile and the top is the 75th percentile, horizontal lines correspond to the median, and whiskers indicate maximum observed values plus +1.5 interquartile range. Black dots are outliers.

As in 2003 [32], we found a positive and significant relationship between forest fragment area and plant species richness in 2013 (R^2^ = 0.28, P < 0.0001, Fig 2, Table 2). Because there was no interaction between year and fragment area (z = 1.06; P = 0.288), the slopes observed in 2003 and 2013 for the relationship of fragment area and species richness are similar (Fig 2). There was no difference in total species richness between forest edge and interior throughout the fragmentation-size gradient in both years (z = 0.515; P = 0.606) indicating that position within the fragment did not affect species richness in any year. Similarly, there was no interaction between species life form and fragment area (z = 1.21; P = 0.225) indicating that woody and non-woody species richness decreased at the same rate with decreasing fragment area. We found a significant interaction between year and species life form: in 2003 there was higher richness of non-woody than woody species; however, in 2013 both woody and non-woody species richness decreased, reaching similar species richness (Table 2, Fig 3). Such result indicates that non-woody, short-lived species richness was more strongly reduced than woody, long-lived species richness in 2013 (Fig 3). This is also observed by the relative percentage of remaining species, where 80% of woody but only 44% of non-woody species remained from 2003 to 2013 (S1 Table).

**Table 2:** Results of generalized linear mixed effects models on species richness. Effects of fragment area, year (2003–2013), and life form (woody and non-woody) on plant species richness in 18 Chaco Serrano forest fragments. We report the effects and *z* values estimated from the reduced model.

Source of variation	Estimated effects (SE)	z values
Log10(Fragment area)	0.13 (0.02)	5.42 ***
Year	-0.27 (0.05)	-5.3 ***
Life form	0.25 (0.04)	5.77 ***
Life form x Log10(fragment area)	-0.30 (0.07)	-4.34 ***

*** P≤0.0001

** P≤0.001

* P≤0.01

SE: Standard error

**Fig 4:**
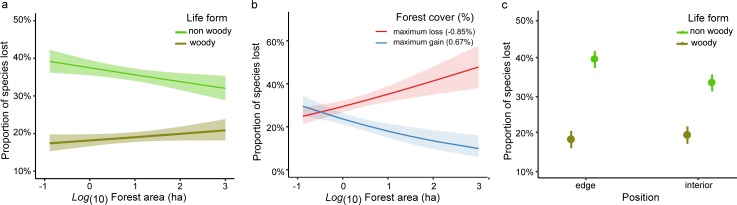
Proportion of species lost. Predicted probabilities and 95% confidence intervals for the marginal effect of three interaction terms on the proportion of species lost. a) Marginal effect of the log_10_ of forest fragment area in relation to life form (non woody- woody), b) Marginal effect of the log_10_ of forest fragment area in relation to maximum loss (red) and gain (blue) values of percent change in forest cover, c) Marginal effect of the position within the fragment (edge-interior) in relation to life form (non woody- woody).

For the proportion of species lost, life form interacted with fragment area indicating that non-woody species were lost in a larger proportion than woody species as the area of forest fragments decreased, but such difference between the proportion of lost woody and non-woody species decreased as the area of forests increased (Table 3, Fig 4A). Importantly, for the proportion of species lost, forest fragment area interacted with percent change in surrounding forest cover at 250m and 500m, whereas no interaction was observed at 1000m. In small forest fragments the proportion of species lost was similar regardless whether the surrounding forest cover decreased or increased (c.a. 25% species lost). However, the proportion of species lost in larger fragments increased when surrounding forest cover decreased and vice versa (Table 3, Fig 4B). In other words, surrounding forest cover had less influence in smaller forest fragments whereas in larger forest fragments it depended on the changes occurred in the surroundings (Fig 4B). Moreover, non-woody species were lost in larger proportion at the edges than the interiors, whereas woody species were lost in similarly lower proportions at both positions (Table 3, Fig 4C). Likewise, non-woody species were also gained in higher proportion than woody species (Table 3, S1 Fig) irrespective of forest remnant area (z = 0.303; P = 0.762), surrounding forest cover at 250m or 500m (z ⋜ 0.808; P ⋝ 0.255), and position (z = 1.265; P = 0.206). The overall proportion of gained species was larger at the edges than the interior across the gradient and irrespective of the statistical model used (Table 3, S1 Fig).

**Table 3:** Results of generalized linear mixed effects models on the proportion of species lost and gained. Effects of fragment area, percent change of surrounding forest cover at 250m and 500m, life form (woody and non-woody), position (edge-interior), and year (2003–2013) on the proportion of lost and gained species in 18 Chaco Serrano forest fragments. We report the effects and *z* values estimated from the reduced model. Reduced models for the proportion of gained species were similar considering forest cover at 250m and 500m and only results at 250m are shown. No interaction was observed at 1000m for lost and gained species, thus we do not show these results.

Proportion of species lost			
Model 1	Source of variation	Estimated effects (SE)	z values
Percent change in forest cover at 250m	Log10(Fragment area)	-0.14 (0.04)	-2.78 **
	% change in forest cover at 250m	-0.19 (0.11)	-1.76 *
	Position	-0.23 (0.08)	-2.75 **
	Life form	-1.12 (0.09)	-12.70 ***
	Life form x Log10(Fragment area)	0.13 (0.04)	-2.94 **
	Position x Life form	0.26 (0.10)	2.36 *
	% change in forest cover at 250m x Log10(Fragment area)	-0.40 (0.14)	-2.78 **
Model 2			
Percent change in forest cover at 500m	Log10(Fragment area)	-0.14 (0.05)	-2.94**
	% change in forest cover at 500m	-0.12 (0.12)	-0.97n.s.
	Position	-0.24 (0.08)	-2.76**
	Life form	-1.12 (0.09)	-12.70***
	Life form x Log10(Fragment area)	0.14 (0.05)	2.93**
	Position x Life form	0.26 (0.11)	2.36*
	% change in forest cover at 500m x Log10(Fragment area)	-0.41 (0.14)	-2.96**
Proportion of species gained			
Model 1	Source of variation	Estimated effects (SE)	z values
Percent change in forest cover at 250m	Position	-0.25 (0.12)	-2.17*
	life form	-0.38 (0.06)	-5.92***

*** P≤0.0001

** P≤0.001

* P≤0.01

SE: Standard error

## Discussion

Habitat loss and fragmentation in the Chaco Serrano forest of central Argentina began to take place at large scales in the 1970s, when forest cover was replaced by crops and urbanized areas [15–18, 32, 37]. The first study evaluating habitat fragmentation effects on plant species richness in the area was conducted in 2003 [32], and this was our baseline to assess changes in plant species richness. The positive and significant species-area relationship found by Cagnolo et al. [32] implies that a relaxation period had already begun by 2003 when extinction debt had started to be paid [6]. While in 2013 the remaining 18 studied forest fragments preserved the same area there was variation in forest cover surrounding these forest fragments. At a regional scale, across all studied sites, there was a net loss of forest cover after this time period. The overall outcome observed was a net loss of 24% of the species at the regional level, constituting an unprecedented magnitude and rate of plant species loss after only a decade.

While there have been a few studies comparing plant species richness at two different times to assess the occurrence of delayed extinctions [21–26], there is only one study in fragmented landscapes comparable to ours. By using baseline vegetation surveys from the early 1950s in temperate Wisconsin forests, Rogers et al. [25] resurveyed part of these forest stands in 2005 to assess changes in native understory plant richness. They found that the nonsignificant relationship between plant species richness and patch size in 1950 became positive and significant by 2005. They observed an extinction debt payment of 15% of understory plant species after 55 years. Comparing the relative loss of species within a decade, we found a nearly ten-fold larger magnitude and rate of species loss in a subtropical seasonally-dry fragmented forest, representing the first report in this understudied forest community of South America [13, 14].

Contrary to our initial expectation of increased slope in plant species richness-area in 2013, we found that the magnitude of species loss was similar throughout the fragmentation size gradient, thus small and large forest fragments lost similar number of species. The results observed here may be explained by the significant interaction between fragment area and percent change in surrounding forest cover at 250m and 500m from the studied forest fragments for the proportion of species lost. This interaction indicates that changes in the surrounding forest cover had less influence in smaller forest fragments than in larger ones. In larger forest fragments the proportion of species lost inversely mirrored the pattern of surrounding forest cover changes after a decade: there were high or low proportions of species lost depending on whether the amount of forest cover decreased or increased, respectively. In contrast, small forest fragments showed a relatively constant proportion of species lost (~25%) regardless of either forest cover increased or decreased in their surroundings. These results suggest an overall degradation in habitat quality within small forest remnants and in their surrounding buffer areas. In other words, there is such a low proportion of forest cover left surrounding these small forest fragments that the changes observed do not appear to have any impact on their connectivity. Notice that in the case of larger forest remnants (> 10.7ha), the circular buffer areas of 250m and 500m imply that at least part of the changes in forest cover (shrub and tree cover) is taking place within them. Hence, while the area of these forest fragments remained as in 2003, there was an impoverishment of high vegetation cover at local and regional levels across this time period. In this regard, there are two important anthropogenic drivers that may be affecting habitat quality within the studied forest remnants and in the surrounding buffer areas. Cattle production is generally in a relatively low-density but there are literally no single cattle-free forest remnants within the studied region [16, 35, 50, 51]. Grazing and trampling by domestic cattle can seriously affect the growth and recruitment of most plant species, as it has been observed in the studied region [50, 51]. Secondly, the Chaco Serrano presents the highest numbers of fire events, burned area, and fire frequency in central Argentina over the past 18 years [52], which are mainly caused by humans due to negligent ignitions for cattle pasture management [52, 53]. We argue that both of these anthropogenic drivers have probably had stronger influence in the forest cover of larger than smaller forest remnants due to their traditional different land uses. While most of the smaller forest fragments studied currently remains embedded in highly modified agricultural matrices, the larger forest fragments are mostly subjected to cattle production, which in turn are also more frequently burnt [52, 53]. These different land uses may help explain the forest cover patterns changes observed across the studied forest remnants after a decade and therefore help to understand their similar species loss across the fragmentation-size gradient.

After ten years we found a higher proportion of loss and gained species at forest edges, indicating higher species turnover at edges than in forest interiors, in agreement with previous findings in other fragmented forests (e.g. [28, 29]). Environmental variability is higher at forest edges due to the proximity of the structurally dissimilar matrix, and therefore edges are more prone to changes in abiotic conditions such as increased exposure to solar radiation, droughts, and windstorms as well as herbicides and pesticides used in crops [28, 54]. Population growth rates of non-woody short-lived species are particularly sensitive to environmental fluctuations that affect key vital rates such as reproduction and survival, whereas woody long-lived species are comparatively more resilient [55]. On the other hand, because of their higher turnover rates, non-woody short-lived species were able to colonize sites more successfully than woody long-lived species. Finally, non-woody species were also lost in larger proportion as the area of forest fragments decreased. Such result may imply that smaller forest fragments, which ranged from 0.13 to 3.58ha, are subjected to overwhelming edge effects within their entire small areas [28, 54].

There was strong imbalance between species loss and gain at the fragment and regional levels, with a lower proportion of species gain, which was not related to patch area nor percent change in forest cover at any buffer area distance. Such results imply a low functional connectivity among remaining forest fragments where species go extinct much faster than they can colonize them [1, 27, 29]. Our observations of decreased forest cover after a decade match these expectations and concur with other previous patch-based pattern analyses showing a generalized forest cover loss over the last few decades in different sectors of the Chaco forests [15–18]. For example, through multi-scale context analysis Frate et al. [18] recently found that the fragmentation process has escalated over the 1979–2010 time period affecting forest cover and connectivity at local and regional scales in NW Chaco forest regions of Cordoba province. Thus, our results suggest this system has not yet reached a new equilibrium, but it is still going through relaxation time (sensu [6]). Immigration delays of species would only be expected in new or restored forest fragments, but not in stable or decreasing ones [1]. Thus, it is very unlikely to observe higher species immigration in the future unless the area of native forests is increased through active management such as promoting restoration of degraded habitats, new legislation to limiting the expansion of agricultural and urban areas, as well as generating corridors or less hostile crop management [30, 56]. Moreover, higher in situ species regeneration by persistent seed banks seems also unlikely due to the persistent cattle presence and frequent use of fire in the studied Chaco Serrano region [50–53].

The nature of the surrounding landscape strongly influences the quality and resilience of forest remnants [18, 19, 33] affecting the recruitment, survival and long-term viability of plant populations [6]. We show here that despite fragment area remained similar after a decade, habitat quality and availability at the landscape scale is of fundamental importance in shaping the dynamics of species extinction and immigration at any given forest remnant. Our results highlight the conservation value of the surrounding landscape for plant species persistence in forest remnants. If plant species immigration does not significantly increase over the next years, then it is likely that plant extinctions will continue through the coming decades, and species richness will decline to a point where extinctions at the fragment level can become regional species extinctions to the Chaco Serrano, leading not only to changes in the physiognomy of the vegetation, but also to a loss of key ecosystem services.

## Supporting information

S1 Table List of sampled plant species. List of plant species sampled across 19 and 18 forest fragments in 2003 and 2013, respectively. The presence of each plant species is denoted with 1 and the absence with 0. Total number of species is given at the end.
(DOCX)

S1 Fig Proportion of plant species gained after a decade. Proportion of (a) gained woody and non-woody species and of (b) gained plant species in edge and interior after a decade. The bottom of each box is the 25th percentile and the top is the 75th percentile, horizontal lines correspond to the median, and whiskers indicate maximum observed values plus +1.5 interquartile range. Black dots are outliers.
(EPS)
