# Supplementary material for: Unprecedented plant species loss after a decade in fragmented subtropical Chaco Serrano forests
Source: PLoS One. 2018 Nov 28;13(11):e0206738. doi: 10.1371/journal.pone.0206738 (PMC6261552; doi:10.1371/journal.pone.0206738)
Supplement: S1 Table — List of plant species sampled across 19 and 18 forest fragments in 2003 and 2013, respectively. The presence of each plant species is denoted with 1 and the absence with 0. Total number of species is given at the end. (DOCX) [file pone.0206738.s001.docx]

**Table S1.** List of plant species sampled across 19 and 18 forest fragments in 2003 and 2013, respectively. The presence of each plant species is denoted with 1 and the absence with 0. Total number of species is given at the end.

| Taxa | Family | Life form | Present in 2003 | Present in 2013 |
| --- | --- | --- | --- | --- |
| *Abobra tenuifolia* (Gillies ex Hook. & Arn.) Cogn. | Cucurbitaceae | non woody | 0 | 1 |
| *Abutilon grandifolium* (Willd.) Sweet | Malvaceae | non woody | 1 | 1 |
| *Abutilon pauciflorum* A. St.-Hil. | Malvaceae | non woody | 1 | 0 |
| *Acacia aroma* Gillies ex Hook. & Arn. | Fabaceae | woody | 1 | 1 |
| *Acacia atramentaria* Benth. | Fabaceae | woody | 0 | 1 |
| *Acacia caven* (Molina) Molina | Fabaceae | woody | 1 | 1 |
| *Acacia praecox* Griseb. | Fabaceae | woody | 1 | 1 |
| *Acaena pinnatifida* Ruiz & Pav. | Rosaceae | non woody | 1 | 0 |
| *Acalypha communis* Müll. Arg. | Euphorbiaceae | non woody | 1 | 1 |
| *Acanthostyles buniifolius* (Hook. & Arn.) R.M. King & H. Rob. | Asteraceae | non woody | 1 | 0 |
| *Achyrocline flaccida* (Weinm.) DC. | Asteraceae | non woody | 1 | 0 |
| *Achyrocline satureioides* (Lam.) DC. | Asteraceae | non woody | 1 | 0 |
| *Adiantopsis tweediana* (Hook.) Link-Pérez & Hickey | Pteridaceae | non woody | 0 | 1 |
| *Agrostis montevidiensis* Spreng. ex Nees | Poaceae | non woody | 1 | 0 |
| *Aloysia gratissima* (Gillies & Hook. ex Hook.) Tronc. | Verbenaceae | non woody | 1 | 1 |
| *Alternanthera pungens* Kunth | Amaranthaceae | non woody | 1 | 1 |
| *Amaranthus hybridus* L. ssp. *hybridus* | Amaranthaceae | non woody | 1 | 0 |
| *Amphilophium carolinae* (Lindl.) L. G. Lohmann | Bignoniaceae | non woody | 1 | 1 |
| *Anemia tomentosa* (Savigny) Sw. | Anemiaceae | non woody | 1 | 1 |
| *Anoda cristata* (L.) Schltdl. | Malvaceae | non woody | 1 | 0 |
| *Anredera cordifolia* (Ten.) Steenis | Basellaceae | non woody | 1 | 1 |
| *Araujia brachystephana* (Griseb.) Fontella & Goyder | Apocynaceae | non woody | 1 | 1 |
| *Araujia odorata* (Hook. & Arn.) Fontella & Goyder | Apocynaceae | non woody | 1 | 0 |
| *Araujia stuckertiana* (Kurtz ex Heger) Fontella & Goyder | Apocynaceae | non woody | 1 | 0 |
| *Arctium minus* (Hill) Bernh. | Asteraceae | non woody | 1 | 0 |
| *Aristolochia argentina* Griseb. | Aristolochiaceae | non woody | 0 | 1 |
| *Artemisia annua* L. | Asteraceae | non woody | 1 | 0 |
| *Aspidosperma quebraco-blanco* Schltdl. | Apocynaceae | woody | 1 | 1 |
| *Austrobrickellia arnottii* (Baker) R.M. King & H. Rob. | Asteraceae | non woody | 1 | 0 |
| *Austroeupatorium inulifolium* (Kunth) R.M. King & H. Rob. | Asteraceae | non woody | 1 | 0 |
| *Ayenia cordobensis* (Hieron.) Hieron. | Malvaceae | non woody | 1 | 0 |
| *Baccharis aliena* (Spreng.) Joch.Müll. | Asteraceae | woody | 1 | 1 |
| *Baccharis articulata* (Lam.) Pers. | Asteraceae | woody | 1 | 0 |
| *Baccharis coridifolia* DC. | Asteraceae | woody | 1 | 1 |
| *Baccharis flabellata* Hook. & Arn. | Asteraceae | woody | 1 | 1 |
| *Baccharis glutinosa* Pers. | Asteraceae | woody | 1 | 0 |
| *Baccharis salicifolia* (Ruiz & Pav.) Pers. | Asteraceae | woody | 0 | 1 |
| *Berberis ruscifolia* Lam. | Berberidaceae | woody | 1 | 1 |
| *Bidens pilosa* L. | Asteraceae | non woody | 1 | 1 |
| *Bidens subalternans* DC. | Asteraceae | non woody | 1 | 1 |
| *Bothriochloa laguroides* (DC.) Herter | Poaceae | non woody | 1 | 0 |
| *Bougainvillea stipitata* Griseb. | Nyctaginaceae | woody | 1 | 1 |
| *Bouteloua curtipendula* (Michx.) Torr. var. *caespitosa* Gould & Kapadia | Poaceae | non woody | 1 | 0 |
| *Bowlesia lobata* Ruiz & Pav. | Apiaceae | non woody | 1 | 1 |
| *Bromelia urbaniana* (Mez) L.B. Sm. | Bromeliacae | non woody | 0 | 1 |
| *Bromus catharticus* Vahl | Poaceae | non woody | 1 | 0 |
| *Broussonetia papyrifera* (L.) Vent. | Moraceae | woody | 1 | 1 |
| *Buddleja stachyoides* Cham. & Schltdl. | Scrophulariaceae | non woody | 1 | 0 |
| *Caesalpinia gilliesii* (Wall. ex Hook.) D. Dietr. | Fabaceae | woody | 1 | 1 |
| *Cantinoa mutabilis* (Rich.) Harley & J.F.B. Pastore | Lamiaceae | non woody | 1 | 1 |
| *Capparis atamisquea* Kuntze | Capparaceae | woody | 1 | 0 |
| *Capsella bursa-pastoris* (L.) Medik. | Brassicaceae | non woody | 1 | 1 |
| *Cardiospermum halicacabum* L. | Sapindaceae | non woody | 1 | 1 |
| *Carduus nutans* L. | Asteraceae | non woody | 1 | 1 |
| *Celtis ehrenbergiana* (Klotzsch) Liebm. | Celtidaceae | woody | 1 | 1 |
| *Cenchrus myosuroides* Kunth | Poaceae | non woody | 1 | 1 |
| *Cestrum parqui* L'Hér. | Solanaceae | woody | 1 | 1 |
| *Chaptalia nutans* (L.) Pol. | Asteraceae | non woody | 1 | 1 |
| *Cheilanthes buchtienii* (Rosenst.) R.M. Tryon | Pteridaceae | non woody | 1 | 0 |
| *Chenopodium album* L. | Chenopodiaceae | non woody | 1 | 1 |
| *Chenopodium cordobense* Aellen | Chenopodiaceae | non woody | 1 | 1 |
| *Chevreulia acuminata* Less. | Asteraceae | non woody | 1 | 0 |
| *Chiropetalum griseum* Griseb. | Euphorbiaceae | woody | 1 | 1 |
| *Chloris* gayana Kunth | Poaceae | non woody | 1 | 0 |
| *Chromolaena arnottiana* (Griseb.) R.M. King & H. Rob. | Asteraceae | non woody | 1 | 1 |
| *Chromolaena hookeriana* (Griseb.) R.M. King & H. Rob. | Asteraceae | woody | 1 | 1 |
| *Cichorium intybus* L. | Asteraceae | non woody | 1 | 1 |
| *Cirsium vulgare* (Savi) Ten. | Asteraceae | non woody | 1 | 0 |
| *Clematis montevidensis* Spreng. var. *montevidensis* | Ranunculaceae | woody | 1 | 1 |
| *Colletia spinosissima* J.F. Gmel. | Rhamnaceae | woody | 1 | 1 |
| *Commelina erecta* L. | Commelinaceae | non woody | 1 | 1 |
| *Condalia buxifolia* Reissek | Rhamnaceae | woody | 1 | 1 |
| *Condalia microphylla* Cav. | Rhamnaceae | woody | 1 | 1 |
| *Condalia montana* A. Cast. | Rhamnaceae | woody | 1 | 1 |
| *Conium maculatum* L. | Apiaceae | non woody | 1 | 1 |
| *Conyza bonariensis* (L.) Cronquist | Asteraceae | non woody | 1 | 0 |
| *Coursetia hassleri* Chodat | Fabaceae | non woody | 0 | 1 |
| *Croton hirtus* L'Hér. | Euphorbiaceae | non woody | 1 | 1 |
| *Croton lachnostachyus* Baill. | Euphorbiaceae | non woody | 1 | 1 |
| *Cucurbitella asperata* (Gillies ex Hook. & Arn.) Walp. | Cucurbitaceae | non woody | 1 | 0 |
| *Cuscuta* sp. | Convolvulaceae | non woody | 0 | 1 |
| *Cyclanthera hystrix* (Gillies ex Hook. & Arn.) Arn. | Cucurbitaceae | non woody | 1 | 0 |
| *Cyclopogon elatus* (Sw.) Schltr. | Orchidaceae | non woody | 1 | 0 |
| *Cynodon dactylon* (L.) Pers. | Poaceae | non woody | 1 | 0 |
| *Cyperus aggregatus* (Willd.) Endl. | Cyperaceae | non woody | 1 | 1 |
| *Cyperus incomtus* Kunth | Cyperaceae | non woody | 1 | 1 |
| *Datura ferox* L. | Solanaceae | non woody | 1 | 0 |
| *Daucus pusillus* Michx. | Apiaceae | non woody | 1 | 0 |
| *Descurainia erodiifolia* (Phil.) Prantl ex Reiche | Brassicaceae | non woody | 1 | 0 |
| *Desmanthus acuminatus* Benth. | Fabaceae | non woody | 1 | 0 |
| *Desmodium uncinatum* (Jacq.) DC. | Fabaceae | woody | 1 | 1 |
| *Dichondra microcalyx* (Hallier f.) Fabris | Convolvulaceae | non woody | 1 | 1 |
| *Dichondra sericea* Sw. | Convolvulaceae | non woody | 1 | 0 |
| *Dicliptera squarrosa* Nees | Acanthaceae | non woody | 1 | 1 |
| *Digitaria sanguinalis* (L.) Scop. | Poaceae | non woody | 1 | 1 |
| *Ditassa burchellii* Hook. & Arn. | Apocynaceae | non woody | 1 | 1 |
| *Dolichandra cynanchoides* Cham. | Bignoniaceae | woody | 1 | 1 |
| *Doryopteris concolor* (Langsd. & Fisch.) Kuhn | Pteridaceae | non woody | 1 | 0 |
| *Duchesnea indica* (Andrews) Focke | Rosaceae | non woody | 1 | 1 |
| *Dysphania ambrosioides* (L.) Mosyakin & Clemants | Chenopodiaceae | non woody | 0 | 1 |
| *Eleusine tristachya* (Lam.) Lam. | Poaceae | non woody | 1 | 0 |
| *Ephedra triandra* Tul. emend. J.H. Hunz. | Ephedraceae | woody | 1 | 1 |
| *Eragrostis lugens* Nees | Poaceae | non woody | 1 | 0 |
| *Eragrostis mexicana* (Hornem.) Link | Poaceae | non woody | 1 | 1 |
| *Eryngium horridum* Malme | Apiaceae | non woody | 1 | 1 |
| *Euphorbia acerensis* Boiss. | Euphorbiaceae | non woody | 1 | 1 |
| *Euphorbia berteroana* Spreng. | Euphorbiaceae | non woody | 1 | 1 |
| *Euphorbia dentata* Michx. | Euphorbiaceae | non woody | 1 | 0 |
| *Euphorbia pulcherrima* Willd. ex Klotzsch | Euphorbiaceae | non woody | 0 | 1 |
| *Eustachys retusa* (Lag.) Kunth | Poaceae | non woody | 0 | 1 |
| *Exhalimolobos weddellii* (E. Fourn.) Al-Shehbaz & C.D. Bailey | Brassicaceae | non woody | 1 | 0 |
| *Fleischmannia prasiifolia* (Griseb.) R.M. King & H. Rob. | Asteraceae | non woody | 1 | 1 |
| *Flourensia campestris* Griseb. | Asteraceae | woody | 1 | 0 |
| *Galium latoramosum* Clos | Rubiaceae | non woody | 1 | 1 |
| *Galium richardianum* (Gillies ex Hook. & Arn.) Endl. ex Walp. ssp. *richardianum* | Rubiaceae | non woody | 1 | 1 |
| *Gaya parviflora* (Phil.) Krapov. | Malvaceae | non woody | 0 | 1 |
| *Geoffroea decorticans* (Gillies ex Hook. & Arn.) Burkart | Fabaceae | woody | 1 | 1 |
| *Glandularia dissecta* (Willd. ex Spreng.) Schnack & Covas | Verbenaceae | non woody | 1 | 0 |
| *Glandularia peruviana* (L.) Small | Verbenaceae | non woody | 1 | 1 |
| *Gleditsia triacanthos* L. | Fabaceae | woody | 1 | 1 |
| *Glycine max* (L.) Merr. | Fabaceae | non woody | 1 | 0 |
| *Gomphrena perennis* L. var. *perennis* | Amaranthaceae | non woody | 0 | 1 |
| *Gomphrena pulchella* Mart. | Amaranthaceae | non woody | 1 | 1 |
| *Gouinia latifolia* (Griseb.) Vasey | Poaceae | non woody | 1 | 1 |
| *Heimia salicifolia* (Kunth) Link | Lythraceae | woody | 1 | 1 |
| *Heterosperma ovatifolium* Cav. | Asteraceae | non woody | 1 | 1 |
| *Hieracium palezieuxii* Zahn | Asteraceae | non woody | 1 | 0 |
| *Hirschfeldia incana* (L.) Lagr.-Foss. | Brassicaceae | non woody | 1 | 0 |
| *Ipomoea rubriflora* O'Donell | Convolvulaceae | non woody | 1 | 0 |
| *Ipomoea purpurea* (L.) Roth | Convolvulaceae | non woody | 1 | 1 |
| *Iresine diffusa* Humb. & Bonpl. ex Willd. | Amaranthaceae | non woody | 1 | 1 |
| *Janusia guaranitica* (A. St.-Hil.) A. Juss. | Malpighiaceae | non woody | 1 | 1 |
| *Jarava* sp. | Poaceae | non woody | 1 | 1 |
| *Jodina rhombifolia* (Hook. & Arn.) Reissek | Cervantesiaceae | woody | 1 | 1 |
| *Krapovickasia flavescens* (Cav.) Fryxell | Malvaceae | non woody | 1 | 0 |
| *Lantana balansae* Briq. | Verbenaceae | non woody | 1 | 1 |
| *Lantana camara* L. | Verbenaceae | non woody | 1 | 1 |
| *Lantana grisebachii* Stuck. ex Seckt | Verbenaceae | non woody | 1 | 1 |
| *Leonurus japonicus* Houtt. | Lamiaceae | non woody | 1 | 1 |
| *Lepechinia floribunda* (Benth.) Epling | Lamiaceae | non woody | 1 | 1 |
| *Lessingianthus mollissimus* (D. Don ex Hook. & Arn.) H. Rob. | Asteraceae | non woody | 1 | 0 |
| *Ligustrum lucidum* W.T. Aiton | Oleaceae | woody | 1 | 1 |
| *Lippia junelliana* (Moldenke) Tronc. | Verbenaceae | non woody | 0 | 1 |
| *Lippia turbinata* Griseb. | Verbenaceae | woody | 1 | 1 |
| *Lithraea molleoides* (Vell.) Engl. | Anacardiaceae | woody | 1 | 1 |
| *Lorentzianthus viscidus* (Hook. & Arn.) R.M. King & H. Rob. | Asteraceae | non woody | 1 | 1 |
| *Lycium cestroides* Schltdl. | Solanaceae | woody | 1 | 1 |
| *Lycium ciliatum* Schltdl. | Solanaceae | woody | 1 | 1 |
| *Malvastrum coromandelianum* (L.) Garcke | Malvaceae | non woody | 1 | 1 |
| *Malvastrum interruptum* K. Schum. | Malvaceae | non woody | 1 | 0 |
| *Mandevilla laxa* (Ruiz & Pav.) Woodson | Apocynaceae | woody | 1 | 1 |
| *Mandevilla pentlandiana* (A. DC.) Woodson | Apocynaceae | woody | 1 | 1 |
| *Manihot grahamii* Hook. | Euphorbiaceae | woody | 1 | 1 |
| *Maytenus spinosa* (Griseb.) Lourteig & O'Donell. | Celastraceae | woody | 1 | 1 |
| *Melia azedarach* L. | Meliaceae | woody | 1 | 1 |
| *Melochia argentina* R.E. Fr. | Malvaceae | non woody | 1 | 0 |
| *Mikania urticifolia* Hook. & Arn. | Asteraceae | non woody | 1 | 0 |
| *Minthostachys verticillata* (Griseb.) Epling | Lamiaceae | non woody | 0 | 1 |
| *Modiolastrum malvifolium* (Griseb.) K. Schum. | Malvaceae | non woody | 1 | 0 |
| *Morus alba* L. | Moraceae | woody | 1 | 1 |
| *Myriopteris myriophylla* (Desv.) J. Sm. | Pteridaceae | non woody | 1 | 0 |
| *Nassella cordobensis* (Speg.) Barkworth | Poaceae | non woody | 1 | 0 |
| *Nassella neesiana* (Trin. & Rupr.) Barkworth | Poaceae | non woody | 1 | 0 |
| *Nassella tenuissima* (Trin.) Barkworth | Poaceae | non woody | 1 | 1 |
| *Oenothera indecora* Cambess. | Onagraceae | non woody | 1 | 0 |
| *Ophryosporus axilliflorus* (Griseb.) Hieron. | Asteraceae | non woody | 1 | 1 |
| *Oplismenus hirtellus* (L.) P. Beauv. ssp. *hirtellus* | Poaceae | non woody | 1 | 1 |
| *Opuntia ficus-indica* (L.) Mill. | Cactaceae | woody | 1 | 0 |
| *Opuntia megapotamica* Arechav. | Cactaceae | woody | 1 | 1 |
| *Opuntia salmiana* Parm. | Cactaceae | woody | 1 | 1 |
| *Opuntia sulphurea* Gillies ex Salm-Dyck | Cactaceae | woody | 1 | 1 |
| *Oxalis conorrhiza* Jacq. | Oxalidaceae | non woody | 1 | 1 |
| *Pappophorum pappiferum* (Lam.) Kuntze | Poaceae | non woody | 1 | 0 |
| *Parietaria debilis* G. Forst. | Urticaceae | non woody | 1 | 1 |
| *Paronychia setigera* (Gillies ex Hook. & Arn.) F. Herm. | Caryophyllaceae | non woody | 1 | 0 |
| *Parthenium hysterophorus* L. | Asteraceae | non woody | 1 | 0 |
| *Pascalia glauca* Ortega | Asteraceae | non woody | 0 | 1 |
| *Paspalum malacophyllum* Trin. | Poaceae | non woody | 1 | 1 |
| *Paspalum notatum* Flüggé | Poaceae | non woody | 1 | 0 |
| *Passiflora caerulea* L. | Passifloraceae | non woody | 1 | 1 |
| *Passiflora mooreana* Hook. f. | Passifloraceae | non woody | 0 | 1 |
| *Passiflora morifolia* Mast. | Passifloraceae | non woody | 1 | 1 |
| *Passiflora suberosa* L. | Passifloraceae | non woody | 1 | 1 |
| *Pavonia glechomoides* A. St.-Hil. | Malvaceae | non woody | 1 | 0 |
| *Pavonia hastata* Cav. | Malvaceae | non woody | 1 | 0 |
| *Pavonia revoluta* Krapov. & Cristóbal | Malvaceae | non woody | 0 | 1 |
| *Philibertia gilliesii Hook. & Arn.* | Apocynaceae | non woody | 1 | 1 |
| *Phyllanthus niruri* L. | Phyllantaceae | non woody | 1 | 0 |
| *Piptochaetium montevidense* (Spreng.) Parodi | Poaceae | non woody | 1 | 0 |
| *Plantago major* L. | Plantaginaceae | non woody | 1 | 0 |
| *Pleopeltis pinnatifida* Gillies ex Hook. & Grev. | Polypodiaceae | non woody | 1 | 0 |
| *Plumbago caerulea* Kunth | Plumbaginaceae | non woody | 0 | 1 |
| *Pombalia serrata* (Phil.) Paula-Souza | Violaceae | non woody | 1 | 0 |
| *Porlieria microphylla* (Baill.) Descole, O´Donell & Lourteig | Zygophyllaceae | woody | 1 | 1 |
| *Porophyllum ruderale* (Jacq.) Cass. | Asteraceae | non woody | 0 | 1 |
| *Portulaca grandiflora* Hook. | Portulaceae | non woody | 1 | 0 |
| *Portulaca oleracea* L. | Portulaceae | non woody | 1 | 0 |
| *Prosopis alba* Griseb. | Fabaceae | woody | 1 | 1 |
| *Prosopis chilensis* (Molina) Stuntz emend. Burkart | Fabaceae | woody | 1 | 1 |
| *Prosopis nigra* (Griseb.) Hieron. | Fabaceae | woody | 1 | 1 |
| *Prosopis pugionata* Burkart | Fabaceae | woody | 1 | 1 |
| *Pseudabutilon pedunculatum* (R.E. Fr.) Krapov. | Malvaceae | non woody | 1 | 1 |
| *Pseudabutilon virgatum* (Cav.) Fryxell | Malvaceae | non woody | 1 | 1 |
| *Rhynchosia edulis* Griseb. | Fabaceae | woody | 1 | 1 |
| *Rivina humilis* L. | Phytolaccaceae | non woody | 1 | 1 |
| *Ruprechtia apetala* Wedd. | Polygonaceae | woody | 1 | 1 |
| *Salpichroa origanifolia* (Lam.) Baill. | Solanaceae | non woody | 1 | 1 |
| *Schinus fasciculatus* (Griseb.) I.M. Johnst. | Anacardiaceae | woody | 1 | 1 |
| *Schinus longifolius* (Lindl.) Speg. | Anacardiaceae | woody | 1 | 1 |
| *Schizachyrium microstachyum* (Desv. ex Ham.) Roseng., B.R. Arrill. & Izag. | Poaceae | non woody | 1 | 0 |
| *Schkuhria pinnata* (Lam.) Kuntze ex Thell. | Asteraceae | non woody | 1 | 1 |
| *Senecio pampeanus* Cabrera | Asteraceae | non woody | 1 | 1 |
| *Senna aphylla* (Cav.) H.S. Irwin & Barneby | Fabaceae | woody | 1 | 1 |
| *Senna corymbosa* (Lam.) H.S. Irwin & Barneby | Fabaceae | woody | 1 | 1 |
| *Senna morongii* (Britton) H.S. Irwin & Barneby | Fabaceae | woody | 1 | 0 |
| *Setaria* spp. 1 | Poaceae | non woody | 1 | 1 |
| *Setaria* spp. 2 | Poaceae | non woody | 1 | 1 |
| *Sida dictyocarpa* Griseb. ex K. Schum. | Malvaceae | non woody | 1 | 1 |
| *Sida rhombifolia* L. | Malvaceae | non woody | 1 | 1 |
| *Sida spinosa* L. | Malvaceae | non woody | 1 | 0 |
| *Solanum argentinum* Bitter & Lillo | Solanaceae | non woody | 1 | 1 |
| *Solanum chacoense* Bitter | Solanaceae | non woody | 1 | 0 |
| *Solanum chenopodioides* Lam. | Solanaceae | non woody | 1 | 1 |
| *Solanum palinacanthum* Dunal | Solanaceae | non woody | 0 | 1 |
| *Solanum pseudocapsicum* L. | Solanaceae | non woody | 1 | 0 |
| *Solanum salicifolium* Phil. | Solanaceae | non woody | 1 | 0 |
| *Solanum sisymbriifolium* Lam. | Solanaceae | non woody | 1 | 0 |
| *Solanum stuckertii* Bitter | Solanaceae | non woody | 1 | 1 |
| *Solidago chilensis* Meyen | Asteraceae | non woody | 0 | 1 |
| *Sonchus oleraceus* L. | Asteraceae | non woody | 1 | 0 |
| *Sorghum halepense* (L.) Pers. | Poaceae | non woody | 1 | 0 |
| *Sphaeralcea cordobensis* Krapov. | Malvaceae | non woody | 1 | 0 |
| *Sporobolus indicus* (L.) R. Br. | Poaceae | non woody | 1 | 0 |
| *Sporobolus pyramidatus* (Lam.) Hitchc. | Poaceae | non woody | 1 | 0 |
| *Stenandrium diphyllum* Nees | Acanthaceae | non woody | 1 | 0 |
| *Stevia achalensis* Hieron. | Asteraceae | non woody | 1 | 0 |
| *Stevia satureiifolia* (Lam.) Sch. Bip. ex Klotzsch | Asteraceae | non woody | 1 | 1 |
| *Talinum paniculatum* (Jacq.) Gaertn. | Talinaceae | non woody | 1 | 1 |
| *Taraxacum officinale* G. Weber ex F.H. Wigg. | Asteraceae | non woody | 1 | 0 |
| *Tillandsia capillaris* Ruiz & Pav. | Bromeliaceae | woody | 1 | 1 |
| *Tillandsia castellanii* L.B. Sm. | Bromeliaceae | woody | 1 | 1 |
| *Tragia geraniifolia* Klotzsch ex Baill. | Euphorbiaceae | non woody | 1 | 0 |
| *Tragia volubilis* L. | Euphorbiaceae | non woody | 1 | 0 |
| *Trichloris crinita* (Lag.) Parodi | Poaceae | non woody | 1 | 0 |
| *Trichocline reptans* (Wedd.) Hieron. | Asteraceae | non woody | 1 | 0 |
| *Trixis divaricata* (Kunth) Spreng. ssp. *discolor* (D. Don) Katinas | Asteraceae | non woody | 1 | 1 |
| *Ulmus parvifolia* Jacq. | Ulmaceae | woody | 1 | 1 |
| *Verbena litoralis* Kunth | Verbenaceae | non woody | 1 | 1 |
| *Verbesina encelioides* (Cav.) Benth. & Hook. f. ex A. Gray | Asteraceae | non woody | 1 | 0 |
| *Vernonanthura nudiflora* (Less.) H. Rob. | Asteraceae | non woody | 0 | 1 |
| *Wissadula gymnanthemum* (Griseb.) K. Schum. | Malvaceae | non woody | 0 | 1 |
| *Ximenia americana* L. | Olacaceae | woody | 0 | 1 |
| *Zanthoxylum coco* Gillies ex Hook. f. & Arn. | Rutaceae | woody | 1 | 1 |
| *Zinnia peruviana* (L.) L. | Asteraceae | non woody | 1 | 1 |
| Total number of species | | | 229 | 163 |
